# Supplementary figures and images for: Compounds from medicinal plants produced in hairy root and transgenic hairy root cultures: a review
Source: PeerJ. 2025 Sep 19;13:e19967. doi: 10.7717/peerj.19967 (PMC12452944; doi:10.7717/peerj.19967)

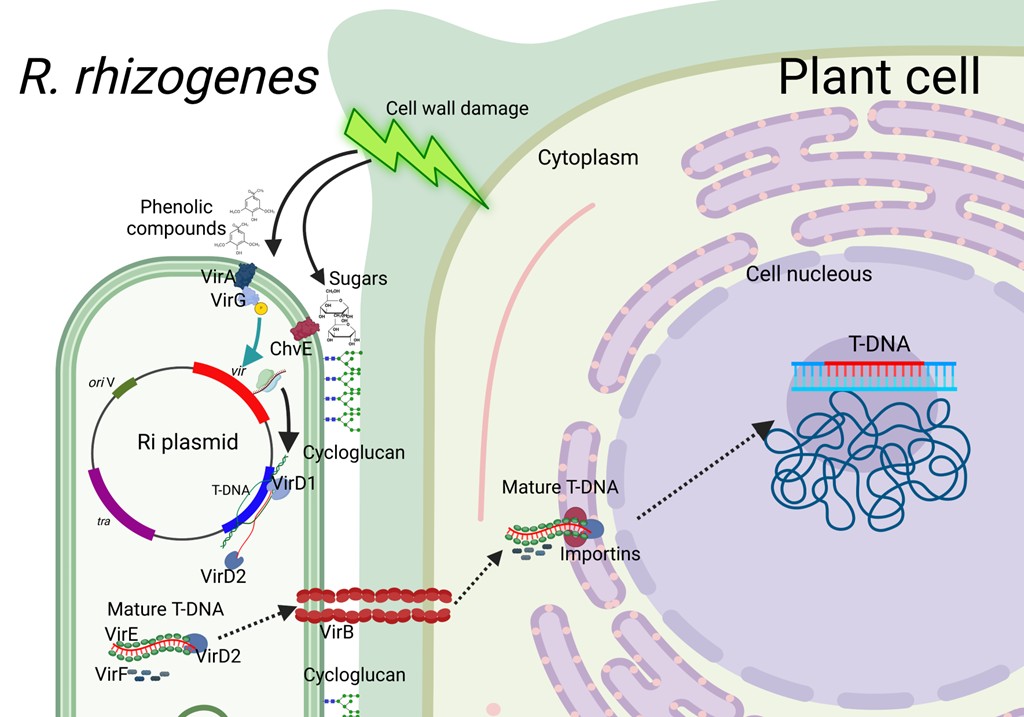

Supplement: Supplemental Information 1 — External black arrows refer to plant cell response to any damage. Phenolics and sugars liberated are recognized by transmembrane VirA protein-bacteria. Then, VirG protein is phosphorylated and cycloglucan is produced to anchor bacteria to plant cell. Green-blue arrow indicates the up-expression level of vir genes stimulated by VirG protein phosphorylation. Black arrow in the bacteria refers to VirD proteins, which promote separation of the double strand DNA. Then, VirD2 binds to the 5’ end and VirD1 binds to the 3’ end of the newly formed strand. When VirE and VirF proteins bind to T-DNA it is referred to as mature T-DNA. Discontinue arrows indicate the mature T-DNA movement from bacteria into cytoplasm and nucleus plant cell. [file peerj-13-19967-s001.jpg]
