# Supplementary material for: Compounds from medicinal plants produced in hairy root and transgenic hairy root cultures: a review
Source: PeerJ. 2025 Sep 19;13:e19967. doi: 10.7717/peerj.19967 (PMC12452944; doi:10.7717/peerj.19967)
Supplement: Supplemental Information 2 — HR: roots induced by wild R. rhizobium strains by other hand. THR: roots induced by strains modified with binary vectors, raw data. [file peerj-13-19967-s002.pdf]

Table S1. Medicinal phenolic compounds produced by transgenic hairy root cultures.

Plant species	*A. rhizogenes* strains	Compounds	Biologycal activity	References
Scutellaria baicalensis Georgi	R1000+pRNAi-SbCHI	Baicalin, baicalein, and wogonin	Anti-inflammatory, antioxidant, and antitumor	Park *et al.* (2011)
Pueraria candollei Grah. ex. Benth	ATCC 43057	Daidzein and puerarin	Antioxidant	Danphitsanuparn *et al.* (2012)
Decalepis arayalpathra (Joseph & Chandras) Venter	TR105	2-Hydroxy-4-methoxy benzaldehyde (MBALD)	Antimicrobial, antioxidant, and antifungal	Sudha *et al.* (2013)
Dracocephalum moldavica L.	A4	Rosmarinic acid	Antioxidant, antiviral, anti-inflammatory, and antimicrobial	Weremczuk-Jeżyna *et al.* (2013)
Hypericum perforatum L.	A4	Xanthones	Antioxidant, antimicrobial, and cytotoxic	Tusevski *et al.* (2013)
Linum album Kotschy ex Boiss	LBA9402	Podophyllotoxin and 6-methoxy podophyllotoxin	Anticancerogenic and antiviral	Chashmi *et al.* (2013)
Linum narbonense L.	ATCC 15834	Justicidine B	Management of bone cancer and osteoclastogenesis	Ionkova *et al.* (2013)
Hyptis suaveolens (L.) Poit	ATCC 15834 and ATCC 15834+pTDT	Podophyllotoxin, 6-methoxy podophyllotoxin, and *β*-peltatin	Anticancerogenic	Bazaldúa *et al.* (2014)
Leontopodium nivale ssp. *alpinum* (Cass.) Greuter	ATCC 15834	Leoligin and 5-methoxy-leoligin	Pro-angiogenic, pro-arteriogenic, and treatment of myocardial infarction	Wawrosch *et al.* (2014)
Liunm mucronatum ssp. mucronatum	A13	Podophyllotoxin and 6-methoxy podophyllotoxin	Anticancerogenic and antiviral	Samadi *et al.* (2014)
Momordica charantia L.	KCTC 2703	Polyphenols	Antioxidant and antimicrobial	Thiruvengadam *et al.* (2014a)
Polygonum multiflorum Thunb	KCTC 2703	Emodin, physcion, and polyphenols	Antioxidant and antimicrobial	Thiruvengadam *et al.* (2014b)
Cucumis anguria L.	KCTC 2703	Flavonols, hydroxycinnamic, and hydroxybenzoic acids	Antioxidant and antimicrobial	Yoon *et al.* (2015)

Table S1. (continued)

Plant species	*A. rhizogenes* strains	Compounds	Biologycal activity	References
Linum album Kotschy ex Boiss and *Linum flavum* L.	LBA 9402 ATCC 15834	Podophyllotoxin, 6-methoxy podophyllotoxin, and methoxypodophyllotoxin-6-glucoside	Antitumor	Cong *et al.* (2015)
Brassica rapa ssp. *rapa* L.	KCTC 2703	Glucosinolates and polyphenols	Antioxidant, antimicrobial, and anticancerogenic	Chung *et al.* (2016)
Fagopyrum tataricum Gaertn	R1000, R1200, R13333, R15834, R1601, LBA9402, and A4	Phenolic acids, cyanidin 3-*O*-glucoside, and cyanidin 3-*O*-rutinoside	Antioxidant, anticancerogenic, and anti-hypertension	Thwe *et al.* (2016)
Linum album Kotschy ex Boiss	LBA9402	Podophyllotoxin, 6-methoxy podophyllotoxin, and phenolic acids	Anticancerogenic and antiviral	Tashackori *et al.*, (2016)
Linum usitatissimum L.	A4	Secoisolariciresinol diglucoside, secoisolariciresinol, and matairesinol	Antioxidant and antitumor	Gabr *et al.* (2016a)
Oldenlandia umbellata L.	MTCC 532	Anthraquinones	Treatment of kidney andbladder stones	Saranya & Siril, (2016)
Ocimum basilicum L.	A4	Rosmarinic acid and caffeic acid	Antioxidant	Srivastava *et al.*, (2016)
Salvia wagneriana Polak	ATCC 15834 and 1855 NCPPB	Rosmarinic acid	Antioxidant	Ruffoni *et al.* (2016)
Silybum marianum L.	A4	Silydianin, silybine, cinnamic acid, and *p*-cumaric acid	Antioxidant, hepatoprotective, and hepatoregenerative	Gabr *et al.*, (2016b)
Lactuca serriola L.	AR15834	Phenolic acids and flavonoids	Antioxidant and cytotoxic	El-Esawi *et al.* (2017)
Althea officinalis L.	A4, A13, ATCC 15834, and ATCC 15834 + GUS	Polyphenols	Antimicrobial, antiviral, and antitussive	Tavassoli & Safipour Afshar (2018)
Linum album Kotschy ex Boiss	LBA9402	Podophyllotoxin and 6-methoxy podophyllotoxin	Anticancerogenic and antiviral	Tashackori *et al.* (2018)

Table S1. (continued)

Plant species	*A. rhizogenes* strains	Compounds	Biologycal activity	References
Linum flavum L.	ATCC 15834	Podophyllotoxin, podophyllotoxin-glucoside, 6-methoxy podophyllotoxin, and 6-methoxy podophyllotoxin glucoside	Cytotoxic and antiviral	Renouard *et al.* (2018)
Nitraria schoberi L.	15834 SWISS	Saponins, phenolic acids, pectins, protopectins, and catechin	Antiviral against influenza virus subtypes A(H5N1) and A (H3N2)	Zheleznichenko *et al.* (2018)
Raphanus sativus L.	MTCC 2364	Quercetin and polyphenols	Antioxidant	Balasubramanian *et al.* (2018)
Salvia viridis L.	A4	Caffeic acid derivatives and rosmarinic acid	Antioxidant and anti-inflammatory	Grzegorczyk-Karolak *et al.* (2018)
Sphagneticola calendulacea L.	LBA1334+pCA and MBIA1391Z	Wedelolactone	Anti-hepatotoxic and anticancerogenic	Kundu *et al.* (2018)
Scutellaria lateriflor L.	R1000	Baicalin, baicalein, and wogonin	Antioxidant and anticancerogenic	Tuan *et al.* (2018)
Salvia miltiorrhiza Bunge	ATCC 15834	Phenolic acids and tanshinones	Antioxidant, anticancerogenic, anti-inflammatory, and antibacterial	Xing *et al.* (2018)
Artemisia vulgaris L.	A4	Flavonoids	Antioxidant	Matvieieva *et al.* (2019)
Aster scaber Thunb.	KCTC 2703	Flavonoids, phenolic acids, and resveratrol	Antioxidant, antimicrobial, antidiabetic, anti-inflammatory, and cytotoxic	Ghimire *et al.* (2019)
Gentiana utriculosa L.	A4M70GUS	Decussatin and decussatin-1-*O*-primeveroside	Hepatoprotective	Vinterhalter *et al.* (2019)
Hyptis suaveolens (L.) Poit.	ATCC 15834+pTDT	Podophyllotoxin	Anticancerogenic	Bazaldúa *et al.* (2019)
Lactuca indica L.	R1000	Hydroxycinnamic acids and flavonoids	Antioxidant	Yi *et al.* (2019)
Cichorium intybus L.	*R. rhizogenes* 2659	3,5-dicaffeoylquinic acid	Antioxidant and antibacterial	Bernard *et al.* (2020)

Table S1. **(continued)**

Plant species	*A. rhizogenes* strains	Compounds	Biologycal activity	References
Cucumis anguria L.	R1000	Polyphenols	Antibacterial and antioxidant	Sahayarayan *et al.* (2020)
Echinacea purpurea L.	ATCC 43057	Caffeic acids derivatives	Antibacterial, antiviral, antifungal, and anticancerogenic	Demirci *et al.* (2020)
Echium plantagineum L.	ATCC 15834	Shikonin and acetylshikonin	Anti-inflammatory, antimicrobial, antitumor, and antiviral	Fu *et al.* (2020)
Hypericum sinaicum L.	A4, A4T, A4T-GUS, and LBA1334	Hypericin	Anti-inflammatory and sedative	Khlifa *et al.* (2020)
Mentha spicata L.	A13, R318, A4, GMI 9534, and ATCC 15834	Phenolic acids	Antioxidant and antimicrobial	Yousefian *et al.* (2020)
Ocimum basilicum L.	R1000	Rosmarinic acid	Antioxidant	Kwon *et al.* (2020)
Salvia bulleyana Diels	A4	Hydroxycinnamic acids	Antimicrobial, antioxidant, and anti-inflammatory	Wojciechowska *et al.* (2020)
Althaea officinalis L., *Artemisia vulgaris* L., and *Artemisia tilesii* Ledeb.	A4+pCB124	Flavonoids	Antioxidant	Bohdanovych *et al.* (2021)
Arachis hypogaea L.	ATCC 15834	Arachidin-6	Antioxidant	Gajurel *et al.* (2021)
Gentiana dinarica Beck	A4 M70GUS	Norswertianin-1-*O*-primeveroside and norswertianin	Antibacterial, antifungal, and antioxidant	Vinterhalter *et al.* (2021)
Panax ginseng C.A. Meyer	R1601-PAP1	Anthocyanins	Antioxidant	Jin *et al.* (2021)
Pelargonium sidoides DC.	*A. tumefaciens* $C_{58}C_1$ +pRiA4	Coumarin and phenolic compounds	Antibacterial and antviral	Yousefian *et al.* (2021)
Rhodiola quadrifida (Pall.) Fisch. & C.A. Mey.	A4	Salidroside and rosavin	Hemostatic and antitussive	Stepanova *et al.* (2021)
Rubia yunnanensis Diels	A4	Quinones	Anticancerogenic and antioxidant	Miao *et al.* (2021)

**Table S1.** (continued)				
**Plant species**	**Plant species**	**Plant species**	**Plant species**	**Plant species**
Salvia miltiorrhiza Bunge	C58C1	Phenols	Antioxidant and anti-inflammatory	Xu *et al.* (2021)
Glycyrrhiza inflata Bat.	R1000-pCAMBIA2301-AtMYB12	Licochalcone A and echinatin	Anti-inflammatory, antioxidant, and antimicrobial	Wu *et al.* (2022)
Curcuma longa L.	A4	Curcumin, demethoxycurcumin, and bisdemethoxycurcumin	Anticamcerogenic and anti-inflammatrory	Sandhya & Giri (2022)
Salvia bulleyana Diels	A4	Phenols	Antioxidant, cytotoxic, and antibacterial	Krzemińska *et al.* (2022)
Salvia nemorosa L.	ATCC 15834	Rosmarinic acid	Antioxidant	Khoshsokhan *et al.* (2022)
Sphaeralcea angustifolia (Cav) G. Don	ATCC 15834/pTDT	Scopoletin and sphaeralcic acid	Anti-inflammatrory	Reyes-Pérez *et al.* (2022)
Agastache rugosa Kuntze	R1000	Rosmarinic acid	Antimicrobial	Yeo *et al.* (2023)
Crotalaria ochroleuca G. Don	R1601	Apigenin-6,8-C-diglucoside, luteolin 6-C-glucoside, and apigenin	Antioxidant and anticancerogenic	Blank *et al.* (2023)
Dracocephalum moldavica L.	A4	Caffeic acid	Antioxidant and anti-inflammatory	Weremczuk-Jeżyna *et al.* (2023)
Plumbago auriculata L.	ATCC 15834	Plumbagin	Anti-inflammatory, antiradiation, and anti-rheumatoid arthritis	Zhao *et al.* (2023)
Salvia plebeia R. Br.	R1000	Rosmarinic acid, phenols, and flavonoids	Antioxidant	Choi *et al.* (2023)
Thymus daenensis Celak	ATCC 15834 and A4	Phenols	Antioxidant	Alamholo & Soltani (2023)
Agastache rugosa (Fisch. & C. A. Mey.)	A4	Rosmarinic acid	Antioxidant	Kozłowska *et al.* (2024)
Calendula officinalis L.	A4	Flavonoids	Antioxidant	Matvieieva *et al.* (2024)

REFERENCES

(*Citations not included in the reference list*)

Bernard G, Alves Dos Santos H, Etienne A, Samaillie J, Neut C, Sahpaz S, Hilbert JL, Gagneul D, Jullian N, Tahrioui A, Chevalier S, Rivière C, Rambaud C. 2020. MeJA elicitation of chicory hairy roots promotes efficient increase of 3,5-diCQA accumulation, a potent antioxidant and antibacterial molecule. *Antibiotics* **9:**659 DOI 10.3390/antibiotics9100659.

Blank DE, Demuner AJ, Carvalho JL, Firmino MJ, Figueiredo TS, Souza GS, Otoni WC. 2023. *Agrobacterium rhizogenes*-mediated transformation of *Crotalaria ochroleuca*: production of flavonoids from hairy roots. *Journal of the Brazilian Chemical Society* **34(12):**1898-1908 DOI 10.21577/0103-5053.20230083.

Bohdanovych TA, Shakhovsky AM, Duplij VP, Ratushnyak YI, Kuchuk MV, Poyedinok NL, Matvieieva NA. 2021. Effects of genetic transformation on the antioxidant activity of “hairy” roots of *Althaea officinalis* L., *Artemisia vulgaris* L., and *Artemisia tilesii* Ledeb. *Cytology and Genetics* **55:**531-539 DOI 10.3103/S0095452721060037.

Chashmi NA, Sharifi M, Yousefzadi M, Behmanesh M, Rezadoost H, Cardillo A, Palazon J. 2013. Analysis of 6-methoxy podophyllotoxin and podophyllotoxin in hairy root cultures of *Linum album* Kotschy ex Boiss. *Medicinal Chemistry Research* **22:**745–752 DOI 10.1007/s00044-012-0067-1.

Demirci T, Akçay UÇ, Göktürk Baydar N. 2020. Physical and biochemical differences in *Agrobacterium rhizogenes*-mediated transgenic hairy root lines of *Echinacea purpurea*. *In Vitro Cellular & Developmental Biology – Plant* **56:**875–881 DOI 10.1007/s11627-020-10090-z.

El-Esawi MA, Elkelish A, Elansary HO, Ali HM, Elshikh M, Witczak J, Ahmad M. 2017. Genetic transformation and hairy root induction enhance the antioxidant potential of *Lactuca serriola* L. *Oxidative Medicine and Cellular Longevity* **2017:**5604746 DOI 10.1155/2017/5604746.

Gabr AMM, Mabrok HB, Ghanem KZ, Blaut M, Smetanska I. 2016a. Lignan accumulation in callus and *Agrobacterium rhizogenes*-mediated hairy root cultures of flax (*Linum usitatissimum*). *Plant Cell, Tissue and Organ Culture* **126:**255–267 DOI 10.1007/s11240-016-0995-4.

Gabr AMM, Ghareeb H, El Shabrawi HM, Smetanska I, Bekheet SA. 2016b. Enhancement of silymarin and phenolic compound accumulation in tissue culture of milk thistle using elicitor feeding and hairy root cultures. *Journal of Genetic Engineering and Biotechnology* **14(2):**327–333 DOI 10.1016/j.jgeb.2016.10.003.

Gajurel G, Hasan R, Medina-Bolivar F. 2021. Antioxidant assessment of prenylated stilbenoid-rich extracts from elicited hairy root cultures of three cultivars of peanut (*Arachis hypogaea*). *Molecules* **26(22):**6778 DOI 10.3390/molecules26226778.

Khlifa HD, Klimek-Chodacka M, Baranski R, Combik M, Taha HS. 2020. *Agrobacterium rhizogenes*-mediated transformation of *Hypericum sinaicum* L. for the development of hairy roots containing hypericin. *Brazilian Journal of Pharmaceutical Sciences* **56:**e18327 DOI 10.1590/s2175-97902020000118327.

Kwon DY, Kim YB, Kim JK, Park SU. 2020. Production of rosmarinic acid and correlated gene expression in hairy root cultures of green and purple basil (*Ocimum basilicum* L.). *Preparative Biochemistry and Biotechnology* **51(1):**35–43

DOI 10.1080/10826068.2020.1789990.
Matvieieva N, Bohdanovych T, Belokurova V, Duplij V, Shakhovsky A, Klymchuk D, Kuchuk M. 2024. Variability in growth and biosynthetic activity of *Calendula officinalis* hairy roots. *Preparative Biochemistry & Biotechnology* **55(4):** 381–391
DOI 10.1080/10826068.2024.2418015
Sahayarayan JJ, Udayakumar R, Arun M, Ganapathi A, Alwahibi MS, Aldosari NS, Morgan AMA. 2020. Effect of different *Agrobacterium rhizogenes* strains for *in-vitro* hairy root induction, total phenolic, flavonoids contents, antibacterial and antioxidant activity of (*Cucumis anguria* L.). *Saudi Journal of Biological Sciences* **27(11):**2972-2979
DOI 10.1016/j.sjbs.2020.08.050.
Saranya Krishnan SR, Siril EA. 2016. Induction of hairy roots and over production of anthraquinones in *Oldenlandia umbellata* L.: a dye yielding medicinal plant by using wild type *Agrobacterium rhizogenes* strain. *Indian Journal of Plant Physiology* **21:**271–278
DOI 10.1007/s40502-016-0229-0.
Stepanova A, Malunova M, Salamaikina S, Selimov R, Solov'eva A. 2021. Establishment of *Rhodiola quadrifida* hairy roots and callus culture to produce bioactive compounds. *Phyton-International Journal of Experimental Botany* **90(2)**:543-552
DOI 10.32604/phyton.2021.013223.
Sudha CG, Sherina TV, Anu Anand VP, Reji JV, Padmesh P, Soniya EV. 2013. *Agrobacterium rhizogenes* mediated transformation of the medicinal plant *Decalepis arayalpathra* and production of 2-hydroxy-4-methoxy benzaldehyde. *Plant Cell, Tissue and Organ Culture* **112:**217–226 DOI 10.1007/s11240-012-0226-6.
Thiruvengadam M, Praveen N, Kim EH, Kim SH, Chung IM. 2014b. Production of anthraquinones, phenolic compounds and biological activities from hairy root cultures of *Polygonum multiflorum* Thunb. *Protoplasma* **251:**555–566 DOI 10.1007/s00709-013-0554-3.
Wawrosch C, Schwaiger S, Stuppner H, Kopp B. 2014. Lignan formation in hairy root cultures of edelweiss (*Leontopodium nivale* ssp. *alpinum* (Cass.) Greuter). *Fitoterapia* **97:**219–223 DOI 10.1016/j.fitote.2014.06.008.
Weremczuk-Jeżyna I, Gonciarz W, Grzegorczyk-Karolak I. 2023. Antioxidant and anti-inflammatory activities of phenolic acid-rich extract from hairy roots of *Dracocephalum moldavica*. *Molecules* **28:**6759 DOI 10.3390/molecules28196759.
Wojciechowska M, Owczarek A, Kiss AK, Grąbkowska R, Olszewska MA, Grzegorczyk-Karolak I. 2020. Establishment of hairy root cultures of *Salvia bulleyana* Diels for production of polyphenolic compounds. *Journal of Biotechnology* **318:**10-19
DOI 10.1016/j.jbiotec.2020.05.002.
Xing B, Yang D, Liu L, Han R, Sun Y, Liang Z. 2018. Phenolic acid production is more effectively enhanced than tanshinone production by methyl jasmonate in *Salvia miltiorrhiza* hairy roots. *Plant Cell, Tissue and Organ Culture* **134:**119–129
DOI 10.1007/s11240-018-1405-x.
Xu W, Jin X, Yang M, Xue S, Luo L, Cao X, Zhang C, Qiao S, Zhang C, Li J, Wu J, LV L, Zhao F, Wang N, Tan S, Lyu-Bu AG, Wang C, Wang X. 2021. Primary and secondary

metabolites produced in *Salvia miltiorrhiza* hairy roots by an endophytic fungal elicitor from *Mucor fragilis*. *Plant Physiology and Biochemistry* **160:**404–412 DOI 10.1016/j.plaphy.2021.01.023.
Yousefian S, Lohrasebi T, Farhadpour M, Haghbeen K. 2020. Production of phenolic acids in hairy root cultures of medicinal plant *Mentha spicata* L. in response to elicitors. *Molecular Biology Research Communications* **9(1):**23-34 DOI 10.22099/mbrc.2020.36031.1475.
