# Supplementary material for: Compounds from medicinal plants produced in hairy root and transgenic hairy root cultures: a review
Source: PeerJ. 2025 Sep 19;13:e19967. doi: 10.7717/peerj.19967 (PMC12452944; doi:10.7717/peerj.19967)
Supplement: Supplemental Information 3 — HR: roots induced by wild R. rhizobium strains by other hand. THR: roots induced by strains modified with binary vectors, raw data. [file peerj-13-19967-s003.pdf]

Table S2. **Terpenes of medicinal importance produced by transgenic hairy roots**

Plant species	*A. rhizogenes* strains	Compounds	Biologycal activity	References
Codonopsis lanceolata Trautv.	R1000	Lancemaside A, foetidissimoside A, and aster saponin Hb	Andropause treatment, anti-inflammatory, and antitumor	Kim *et al.* (2011)
Gentiana cruciata L.	A4, 15834, 8196, and R1000	Gentiopicroside, loganic acid, swertiamarin, and sweroside	Cholagogue, hepatoprotective, and wound healing	Hayta *et al.* (2011)
Gentiana macrophylla Pall.	R1000 pRiA4b	Gentiopicroside	Treat diabetes, paralysis, and rheumatism	Wu *et al.* (2011)
Glycyrrhiza inflata Bat.	ATCC 15834	Glycyrrhizin	Antiherpetic	Wongwicha *et al.* (2011)
Picrorhiza kurroa Royle ex Benth.	A4 and PAT405	Picrotin and picrotoxinin	Used in hepatic disorders, gastric troubles, anemia, and asthma	Mishra *et al.* (2011)
Taraxacum officinale L.	A4 and ATCC 15834	Sesquiterpene lactones	Hepatoprotective, anticancerogenic, anti-inflammatory, antimicrobial, and antimalarial	Mahesh & Jeyachandra n (2011)
Rehmannia glutinosa Libosch.	A4	Iridoid glycosides and phenylethanoid glycosides	Hemostatic, anti-inflmmatory, antitumor, and hypoglycemic	Piątczak *et al.* (2012)
Siegesbeckea orientalis L.	A4	Kirenol	Antibacterial	Wang *et al.* (2012)
Calendula officinalis L.	ATCC 15834	Oleanolic acid derivatives	Anti-inflammatory and cytotoxic	Długosz *et al.* (2013)
Gymnema sylvestre R. Br.	KCTC 2703	Gymnemic acid	Antimicrobial, diuretic, and antihypercholestre mic	Nagella *et al.* (2013)
Withania somnifera L.	R1000	Withanolide A, withanone, and withaferin A	Anti-inflammatory, anxiolytic, and antidepressant	Sivanandhan *et al.* (2013)
Bacopa monnieri L. Wettst.	A4, R1000, SA79, MTCC 532, and MTCC 2364	Bacoside A	Memory-facilitating	Bansal *et al.* (2014)

Table S2. (continued)

Plant species	*A. rhizogenes* strains	Compounds	Biologycal activity	References
Gentiana scabra Bunge	ATCC 15834	Iridoids and secoiridoids	Analgesic, anti-inflammatory, antirheumatic, diuretic, and hypoglycemic	Huang *et al.* (2014)
Valeriana officinalis L.	A13	Valerenic acid	Anxiolytic, sedative and, anticonvulsant	Torkamani *et al.* (2014)
Whitania somnifera L.	R1000, MTCC 2364, and MTCC 532	Withaferin A and withanolide A	Anticancerogenic and neuroprotective	Thilip *et al.* (2015)
Artemisia annua L.	LBA 301	Artemisinin	Antimalarial	Patra & Srivastava (2016)
Artemisia pallens Wall. ex DC	NCIM 5140	Artesunate	Animalarial	Pala *et al.* (2016)
Lopezia racemosa Cav.	ATCC 15834/Ptdt	Campesterol	Cytotoxic and anti-inflammatory	Moreno-Anzúrez *et al.* (2017)
Ferula pseudalliacea Rech.f.	ATCC 15834, 1724, A4, LB9402, and Ar318	Farnesiferol B	Antibacterial, anticancerogenic, and antiplasmodial	Khazaei *et al.* (2019)
Trigonella foenum-graecum L. and *Trigonella monantha* C.A.Mey.	ATCC 15834, A4, and wt	Steroidal sapogenins and phytosterols	Antibacterial, antiviral, and anticancerogenic	Kohsari *et al.* (2020)
Astragalus membranaceus Bge.	R1000	Astragalosides	Treatment of diabetes and cardiovascular diseases	Park *et al.* (2021)
Glycyrrhiza uralensis Fisch.	ATCC 15834	Glycyrrhizin	Anticancerogenic and antiviral	Wang *et al.* (2021)
Panax ginseng C.A. Meyer.	A4	Ginsenosides	Antioxidant and neuroprotective	Zhang *et al.* (2021)
Senna obtusifolia L.	A4	Betulinic acid	Anti-inflmmatory and antiviral	Kowalczyk *et al.* (2021)
Centella asiatica L.	A4	Centellosides	Neuroprotective, antioxidant, antitumor, and anti-inflammatory	Alcalde *et al.* (2022)
Physalis minima L.	A4	Withaferin A	Anticancerogenic	Halder & Ghosh (2023)

Table S2. (continued)

Plant species	*A. rhizogenes* strains	Compounds	Biologycal activity	References
Calendula officinalis L.	ATCC 15834	Oleanolic acid derivatives	Anti-inflammatory and cytotoxic	Kamiński *et al.* (2024)
Salvia miltiorrhiza Bunge	A4	Tanshinone and phenolic compounds	Anti-inflammatory and aticancerogenic	Rastegarnejad *et al.* (2024)

REFERENCES

(*Citations not included in the reference list*)

Alcalde MA, Cusido RM, Moyano E, Palazon J, Bonfill M. 2022. Metabolic gene expression and centelloside production in elicited *Centella asiatica* hairy root cultures. *Industrial Crops and Products* **184:**114988 DOI 10.1016/j.indcrop.2022.114988.

Bansal M, Kumar A, Sudhakara Reddy M. 2014. Influence of *Agrobacterium rhizogenes* strains on hairy root induction and 'bacoside A' production from *Bacopa monnieri* (L.) Wettst. *Acta Physiologiae Plantarum* **36:**2793–2801 DOI 10.1007/s11738-014-1650-5.

Hayta S, Gurel A, Akgun IH, Altan F, Ganzera M, Tanyolac B, Bedir E. 2011. Induction of *Gentiana cruciata* hairy roots and their secondary metabolites. *Biologia* **66:**618–625 DOI 10.2478/s11756-011-0076-4.

Kim JA, Kim YS, Choi YE. 2011. Triterpenoid production and phenotypic changes in hairy roots of *Codonopsis lanceolata* and the plants regenerated from them. *Plant Biotechnology Reports* **5:**255–263 DOI 10.1007/s11816-011-0180-5.

Kowalczyk T, Sitarek P, Merecz-Sadowska A, Szyposzyńska M, Spławska A, Gorniak L, Bijak M, Śliwiński T. 2021. Methyl jasmonate effect on betulinic acid content and biological properties of extract from *Senna obtusifolia* transgenic hairy roots. *Molecules* **26(20):**6208 DOI 10.3390/molecules26206208.

Piątczak E, Królicka A, Wielanek M, Wysokińska H. 2012. Hairy root cultures of *Rehmannia glutinosa* and production of iridoid and phenylethanoid glycosides. *Acta Physiologiae Plantarum* **34:**2215–2224 DOI 10.1007/s11738-012-1022-y.

Thilip C, Soundar Raju C, Varutharaju K, Aslam A, Shajahan A. 2015. Improved *Agrobacterium rhizogenes*-mediated hairy root culture system of *Withania somnifera* (L.) Dunal using sonication and heat treatment. *3 Biotech* **5:**949–956 DOI 10.1007/s13205-015-0297-2.

Wu HJ, Wang XX, Li Y, Zhang DG, Zhang B, Wang XY. 2011. Propagation of *Gentiana macrophylla* (Pall) from hairy root explants via indirect somatic embryogenesis and gentiopicroside content in obtained plants. *Acta Physiologiae Plantarum* **33:**2229–2237 DOI 10.1007/s11738-011-0762-4.
