# Supplementary material for: Compounds from medicinal plants produced in hairy root and transgenic hairy root cultures: a review
Source: PeerJ. 2025 Sep 19;13:e19967. doi: 10.7717/peerj.19967 (PMC12452944; doi:10.7717/peerj.19967)
Supplement: Supplemental Information 4 — HR: roots induced by wild R. rhizobium strains by other hand. THR: roots induced by strains modified with binary vectors, raw data. [file peerj-13-19967-s004.pdf]

Table S3. Alkaloids of medicinal importance produced in transgenic hairy root cultures

Plant species	*A. rhizogenes* strains	Compounds	Biologycal activity	References
Camptotheca acuminata Decne.	*A. tumefaciens* $C_{58}C_1$ (pRi A4)	Camptothecin	Antitumoral	Ni *et al.* (2011)
Peganum harmala L.	15834, TR 105, and LBA	β-Carboline alkaloids	Antiseptic	Zayed (2011)
Ophiorrhiza alata Craib	TISTR 1450	Camptothecin	Anitumoral	Ya-ut *et al.* (2011)
Hyoscyamus muticus L.	A4	Hyoscyamine, scopolamine, and tropine	Antispasmodic, analgesic, and sedative	Dehghan *et al.* (2012)
Physalis ixocarpa Brot.	ATCC 15834 and A4	Hyoscamine, atropine, and scopolamine	Antioxidant	Bergier *et al.* (2012)
Portulaca oleracea L.	ATCC 15834	Dopamine	Analgesic, anti-inflammatory, antifungal, and hypoglycemic	Ahmadi Moghadam *et al.* (2014)
Tribulus terrestris L.	AR15834 and GMI9534	ß-Carboline alkaloids	Anti-inflammatory	Sharifi *et al.* (2014)
Atropa belladona L.	ATCC 15834	Scopolamine	Treatment of parkinson's desease	Habibi *et al.* (2015)
Datura metel L.	A4	Atropine	Anticholinergic and antispasmodic	Shakeran *et al.* (2015)
Catharanthus roseus L.	AR15834 and A4	Catharanthine and ajmalicine	Antihypertensive	Benyammi *et al.* (2016)
Catharanthus roseus L.	K599 +p35SGFPGUS	Vincristine, vinblastine, and catharanthine	Antimicrobial	Hanafy *et al.* (2016)
Papaver orientale L.	ATCC 15834, GM, R1000, and C58C1	Morphinan alkaloids	Analgesic and sedative	Hashemi & Naghavi (2016)
Catharanthus roseus L.	pRiA4	Ajmalicine	Antihypertensive	Thakore *et al.* (2017)
Taxus baccata subsp. *wallichiana*	MTCC 532	Taxol	Anticancerogenic	Sahai & Sinha (2020)
Papaver armeniacum L.	C58C1, ATCC 15834, GM, and R1000	Papaverine, noscapine, thebaine, morphine, and codeine	Sedative and anticancerogenic	Sharifzadeh Naeini *et al.* (2021)
Trigonella foenum-graecum L.	ATCC 11325, ATCC 15834, A4, A7, A13, and K599	Trigonelline	Antidiabetic	Tariverdizadeh *et al.* (2021)

Table S3. (continued)

Plant species	*A. rhizogenes* strains	Compounds	Biologycal activity	References
Taxus× *media* var. *Hicksii* Rehd.	LBA 9402	Paclitaxel	Anticancerogenic	Sykłowska-Baranek *et al.* (2022)
Hyoscyamus muticus L.	A4	Hyoscyamine and hyoscine	Antibacterial	Abdelkawy *et al.* (2023)

REFERENCES

(*Citations not included in the reference list*)

Benyammi R, Paris C, Khelifi-Slaoui M, Zaoui D, Belabbassi O, Bakiri N, Meriem Aci M, Harfi B, Malik S, Makhzoum A, Desobry S, Khelifi L. 2016. Screening and kinetic studies of catharanthine and ajmalicine accumulation and their correlation with growth biomass in *Catharanthus roseus* hairy roots. *Pharmaceutical Biology* **54(10):**2033–2043 DOI 10.3109/13880209.2016.1140213.

Bergier K, Kuźniak E, Skłodowska M. 2012. Antioxidant potential of *Agrobacterium*-transformed and non-transformed *Physalis ixocarpa* plants grown *in vitro* and *ex vitro*. *Postępy Higieny i Medycyny Doświadczalnej* **66:**976–982 DOI 10.5604/17322693.1023086.

Sharifzadeh Naeini M, Naghavi MR, Bihamta MR, Sabokdast M, Salehi M. 2021. Production of some benzylisoquinoline alkaloids in *Papaver armeniacum* L. hairy root cultures elicited with salicylic acid and methyl jasmonate. *In Vitro Cellular & Developmental Biology - Plant* **57:**261–271 DOI 10.1007/s11627-020-10123-7.

Tariverdizadeh N, Mohebodini M, Chamani E, Ebadi A. 2021. Iron and zinc oxide nanoparticles: an efficient elicitor to enhance trigonelline alkaloid production in hairy roots of fenugreek. *Industrial Crops and Products* **162:**113240 DOI 10.1016/j.indcrop.2021.113240.

Zayed R. 2011. Efficient *in vitro* elicitation of β-carboline alkaloids in transformed root cultures of *Peganum harmala*. *Bulletin of Faculty of Pharmacy, Cairo University*, **49(1):**7–11 DOI 10.1016/j.bfopcu.2011.07.002.
